# MicroRNA screening identifies a link between NOVA1 expression and a low level of IKAP in familial dysautonomia

**DOI:** 10.1242/dmm.025841

**Published:** 2016-08-01

**Authors:** Mylène Hervé, El Chérif Ibrahim

**Affiliations:** CRN2M-UMR7286, Aix-Marseille Université, CNRS, Faculté de Médecine Nord, Marseille 13344, Cedex 15, France

**Keywords:** Familial dysautonomia, MicroRNA, Stem cell, NOVA1, IKAP, ELP1

## Abstract

Familial dysautonomia (FD) is a rare neurodegenerative disease caused by a mutation in intron 20 of the *IKBKAP* gene (c.2204+6T>C), leading to tissue-specific skipping of exon 20 and a decrease in the synthesis of the encoded protein IKAP (also known as ELP1). Small non-coding RNAs known as microRNAs (miRNAs) are important post-transcriptional regulators of gene expression and play an essential role in the nervous system development and function. To better understand the neuronal specificity of IKAP loss, we examined expression of miRNAs in human olfactory ecto-mesenchymal stem cells (hOE-MSCs) from five control individuals and five FD patients. We profiled the expression of 373 miRNAs using microfluidics and reverse transcription coupled to quantitative PCR (RT-qPCR) on two biological replicate series of hOE-MSC cultures from healthy controls and FD patients. This led to the total identification of 26 dysregulated miRNAs in FD, validating the existence of a miRNA signature in FD. We then selected the nine most discriminant miRNAs for further analysis. The signaling pathways affected by these dysregulated miRNAs were largely within the nervous system. In addition, many targets of these dysregulated miRNAs had been previously demonstrated to be affected in FD models. Moreover, we found that four of our nine candidate miRNAs target the neuron-specific splicing factor NOVA1. We demonstrated that overexpression of miR-203a-3p leads to a decrease of NOVA1, counter-balanced by an increase of IKAP, supporting a potential interaction between NOVA1 and IKAP. Taken together, these results reinforce the choice of miRNAs as potential therapeutic targets and suggest that NOVA1 could be a regulator of FD pathophysiology.

## Abstract (precis)

**Summary:** A miRNA screening conducted in olfactory stem cells from patients links the neuron-specific splicing factor NOVA1 to neurodegeneration in familial dysautonomia.

## INTRODUCTION

Familial dysautonomia (FD, also known as Riley Day syndrome or hereditary sensory and autonomic neuropathy type III; MIM 223900) is an autosomal recessive disorder that occurs with a carrier frequency of 1 in 30 in the Ashkenazi Jewish population (Scott et al., 2010). This disease is characterized by an incomplete development and a progressive depletion of autonomic and sensory neurons (Axelrod et al., 1981; Pearson and Pytel, 1978; Pearson et al., 1978), which results in variable symptoms including insensitivity to pain, lack of overflow tearing, inappropriate blood pressure control, poor oral coordination and gastrointestinal dysmotility (Axelrod, 2004). Despite recent advances in patient management, ∼50% of patients die before the age of 40 years. No cure is currently available for this neurodegenerative disease and treatment is aimed at controlling symptoms and reducing complications.

FD is caused by mutations in the *IKBKAP* gene, which encodes a protein named IKAP (also known as ELP1) (Anderson et al., 2001; Slaugenhaupt et al., 2001). The most common mutation is the T-to-C transition in position 6 of the 5′ splice site (5′ss) of intron 20 (c.2204+6T>C), present in greater than 99.5% of the disease-causing alleles (Anderson et al., 2001; Dong et al., 2002; Slaugenhaupt et al., 2001). This mutation causes a tissue-specific skipping of exon 20 of *IKBKAP* mRNA, which leads to a severely reduced synthesis of IKAP protein in the central and peripheral nervous systems (Cuajungco et al., 2003).

Although the exact function of the IKAP protein is not clearly understood, IKAP was identified as the scaffold protein required to assemble a well-conserved six-protein complex named the holo-Elongator complex (Glatt and Muller, 2013; Hawkes et al., 2002; Xu et al., 2015). The holo-Elongator is known to hold an enzymatic activity to modifying transfer RNA (Huang et al., 2005; Karlsborn et al., 2014a,b), as well as roles in various processes such as transcription elongation of some human genes, exocytosis, actin cytoskeleton regulation, cell motility, genome demethylation and male meiosis (Close et al., 2006; Creppe et al., 2009; Johansson et al., 2008; Lin et al., 2013; Okada et al., 2010; Rahl et al., 2005).

Several studies investigated the molecular signatures of this disease to better understand pathophysiology of FD. Indeed, transcriptional alterations were revealed in FD cells and tissue by analyzing the transcriptome at the genome-wide level, revealing several groups of dysregulated genes involved in neurogenesis, cell migration or actin cytoskeleton regulation (Boone et al., 2012; Cheishvili et al., 2007; Lee et al., 2009; Lefler et al., 2015). Among the candidate genes that might play a role in regulating *IKBKAP* mRNA alternative splicing, *NOVA1*, a gene encoding the neuron-specific splicing factor, was found to be dysregulated in two different FD stem cell models (Boone et al., 2012; Lee et al., 2009).

Two decades ago, a group of small non-coding RNAs, termed microRNAs (miRNAs), were shown to play essential roles in post-transcriptional regulation of gene expression (Pasquinelli, 2012). These 20- to 22-nucleotide RNAs are able to binding the 3′ untranslated region (3′UTR) of several mRNAs to regulate various human biological processes (Inui et al., 2010). In the brain, miRNAs play key roles in cell fate specification, neurite projection and synaptic plasticity. Moreover, some evidence suggests that dysregulation of miRNA activities can be detrimental to neuronal function (Im and Kenny, 2012). Recently, a few studies have investigated the role of miRNAs in neurodegenerative pathologies (Maciotta et al., 2013), but the expression pattern of miRNAs remains unknown in the FD context.

Cellular models represent a precious tool to better understand and investigate the pathophysiological mechanisms underlying a human genetic disease. In the case of an orphan disease like FD, samples are very difficult to obtain. Nevertheless, *in vitro* cellular models that recapitulate molecular alterations in FD are available. Easily accessible cells like fibroblasts can be reprogrammed into undifferentiated, induced pluripotent stem cells (iPSCs), which present self-renewal properties (Takahashi and Yamanaka, 2006). Encouraging results in the modeling of several neurological pathologies have been obtained using iPSCs to propose new treatments (Lee et al., 2012; Yu et al., 2013). Nevertheless, generation and maintenance of iPSCs remains a laborious task. Therefore, the use of other cellular models, which exhibits self-renewal without a reprogramming step, such as human olfactory ecto-mesenchymal stem cells (hOE-MSCs), is an alternative (Mackay-Sim, 2012). These cells are easily obtained and have been shown to be a useful model that recapitulates molecular variations present in many neurodegenerative diseases like FD (Boone et al., 2010; Cook et al., 2011; Fan et al., 2012, 2013; Ghanbari et al., 2004; Kano et al., 2013; Mor et al., 2013; Stewart et al., 2013).

We had previously shown that FD is characterized by the coordinated dysregulation of mRNAs sets (Boone et al., 2012; Mor et al., 2013). In this study, our goal was to examine for a possible imbalance of miRNA expression in FD, and to predict the associated dysregulated biological processes. We characterized the expression profile of miRNAs in FD, which allowed us to identify dysregulated biological pathways linked to the nervous system. Moreover, we confirmed that overexpression of a dysregulated miRNA in FD, miR-203a-3p, induced underexpression of *NOVA1* transcripts and corrected *IKBKAP* mRNA alternative splicing, leading to a concomitant decrease of NOVA1 and increase of IKAP protein expression. Therefore, these results demonstrate a possible link between miR-203a-3p, NOVA1 and IKAP.

## RESULTS

### Initial testing for *IKBKAP* mRNA alternative splicing in hOE-MSCs

**Fig. 1.**
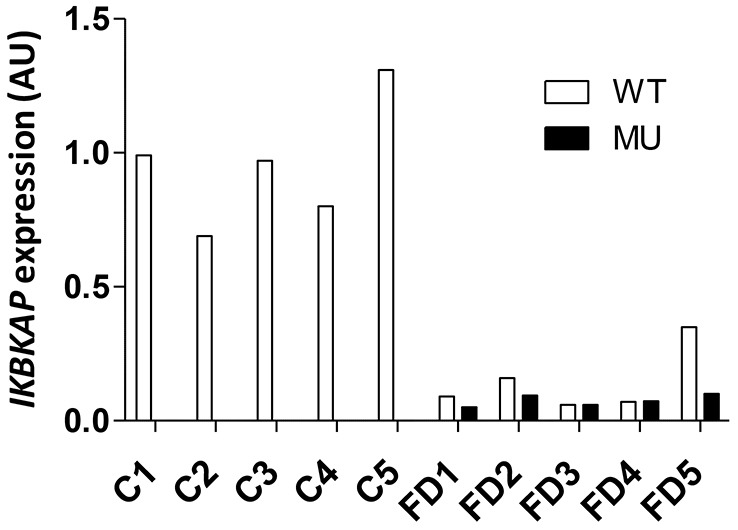
**Expression pattern of *IKBKAP* mRNAs in control and FD hOE-MSCs.** Histograms represent the level of expression of *IKBKAP* alternative transcripts in hOE-MSC cultures for five control (C) and five FD cells, determined by relative RT-qPCR. *WDR59* was used as the reference gene for normalization. The calibrator samples for quantifying WT and MU expression were the mean of control samples and FD samples respectively.

To detect the differential expression of miRNAs in FD compared to control hOE-MSCs, total RNA was extracted from five healthy control and five FD hOE-MSC cultures. To ensure that we would be able to establish links between mRNA and miRNA expression in hOE-MSCs, we first conducted an initial assessment of *IKBKAP* mRNA isoforms by reverse transcription coupled to quantitative PCR (RT-qPCR). As shown on Fig. 1, the *IKBKAP* transcript with exon 20 (WT) is underexpressed by ∼2–25 times compared to control hOE-MSCs in FD cells. Moreover, only FD cells express the *IKBKAP* isofom skipping exon 20 form (called the MU transcript) and they do so to a similar level to the *IKBKAP* WT transcript.

### miRNAs profiling reveals differences between control and FD hOE-MSCs

After a preamplification step in ten hOE-MSC RNA samples previously validated for *IKBKAP* mRNA expression, we conducted the simultaneous amplification of 381 small RNAs, including 373 human miRNAs (Table S1A).

The raw data indicated that a significant proportion (82%) of screened miRNAs were expressed (mean cycle threshold, Ct<40) in our cellular model (Fig. S1A). However, these miRNAs were heterogeneously distributed, and only miRNAs with Ct<32 in >20% of samples were chosen for subsequent analyses. These 253 analyzable miRNAs were clustered into five classes according to their level of expression (Table S2A).

Next, to determine any potential imbalance of miRNAs expression, we identified a list of differentially expressed miRNAs between FD and control hOE-MSCs. After an arbitrary setting of a fold change (FC)≥1.3 and challenging the statistical significance of the microarray results (*P*<0.05) with a parametric Student's *t*-test, eleven miRNAs were found to be dysregulated among FD patients compared to healthy controls, with a similar proportion of miRNAs over- and under-expressed with modest variations (0.54<FC<2.92) (Table 1; Fig. S2).

**Table 1.**
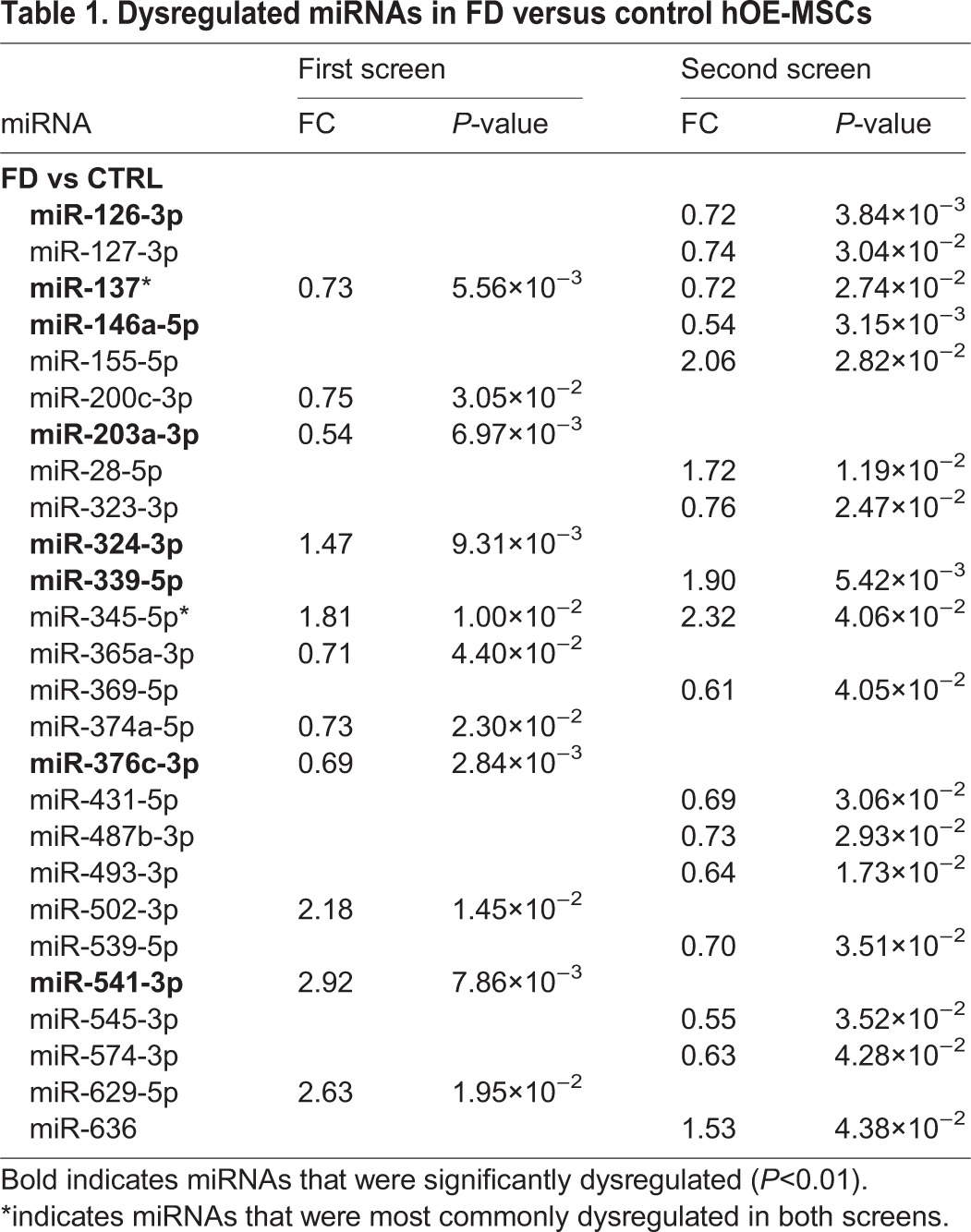
**Dysregulated miRNAs in FD versus control hOE-MSCs**

### Second miRNAs profiling corroborate differences between control and FD cells

To ensure the robustness and reproducibility of our data, we undertook a second miRNA screening with eight biological replicates (different to those above but from controls and patients). We verified the *IKBKAP* mRNA expression pattern in four control and four FD hOE-MSCs, and we found again that the *IKBKAP* WT isoform was underexpressed in FD compared to control cells by ∼6-16 times, and that FD cells expressed the *IKBKAP* MU transcript (Fig. S3). We repeated the previous protocol to simultaneously amplify 381 small RNAs from these eight hOE-MSCs cultures, without the preamplification step owing to limited cell samples (Table S1B).

Similar to what was observed in the first screen, the raw data of our second experiment indicated that an substantial proportion (76%) of screened human miRNA was expressed in our cells (mean Ct<40) (Fig. S1B). As before, because of the heterogeneity of expression, we clustered the 193 analyzable miRNAs (Ct<35 in >25% of samples) in five classes based on their level of expression (Table S2B).

Using the same type of analysis as the first set of samples, we were able to highlight seventeen miRNAs that were dysregulated between FD patients and healthy controls in our biological replicate samples. Modest expression variations (0.54<FC<2.32) were observed along with a predominance of overexpressed miRNAs in FD cells (Table 1; Fig. S2).

To further understand the dysregulated miRNAs in FD pathology and focus on the most promising data, we examined signaling pathways and miRNAs targets for nine of the miRNAs, selecting the two miRNAs most commonly dysregulated in both screens and the seven miRNAs with a *P* value of <0.01 (Table 1, miRNAs in bold).

### Dysregulated miRNAs in FD are associated with neuronal signaling pathways

We next determined the biological pathways related to the nine selected miRNAs differentially expressed between FD and control hOE-MSCs using various databases and algorithms from the DIANA-mirPath v3.0 software (Vlachos et al., 2015b). TarBase v7.0, a database of validated miRNA–gene interactions, in combination with *in silico* predictions using microT-CDS and TargetScan algorithms revealed that various signaling pathways were deficient in FD, and that many of these pathways had already been associated to neuronal and developmental processes in previous studies using different FD modeling paradigms (see references in Table 2 and Table S3). Interestingly, it appeared that one of the dysregulated neuronal pathways was linked to neurotrophins, which are proteins that are known to be essential for differentiation and survival of peripheral neurons (Reichardt, 2006; Tourtellotte, 2016). Moreover, other essential pathways for accurate development of the nervous system seem to be altered in FD pathology, such as the axon guidance and ErbB signaling pathways, with the latter being remarkably associated with peripheral nervous system regeneration (Ronchi et al., 2015).

**Table 2.**
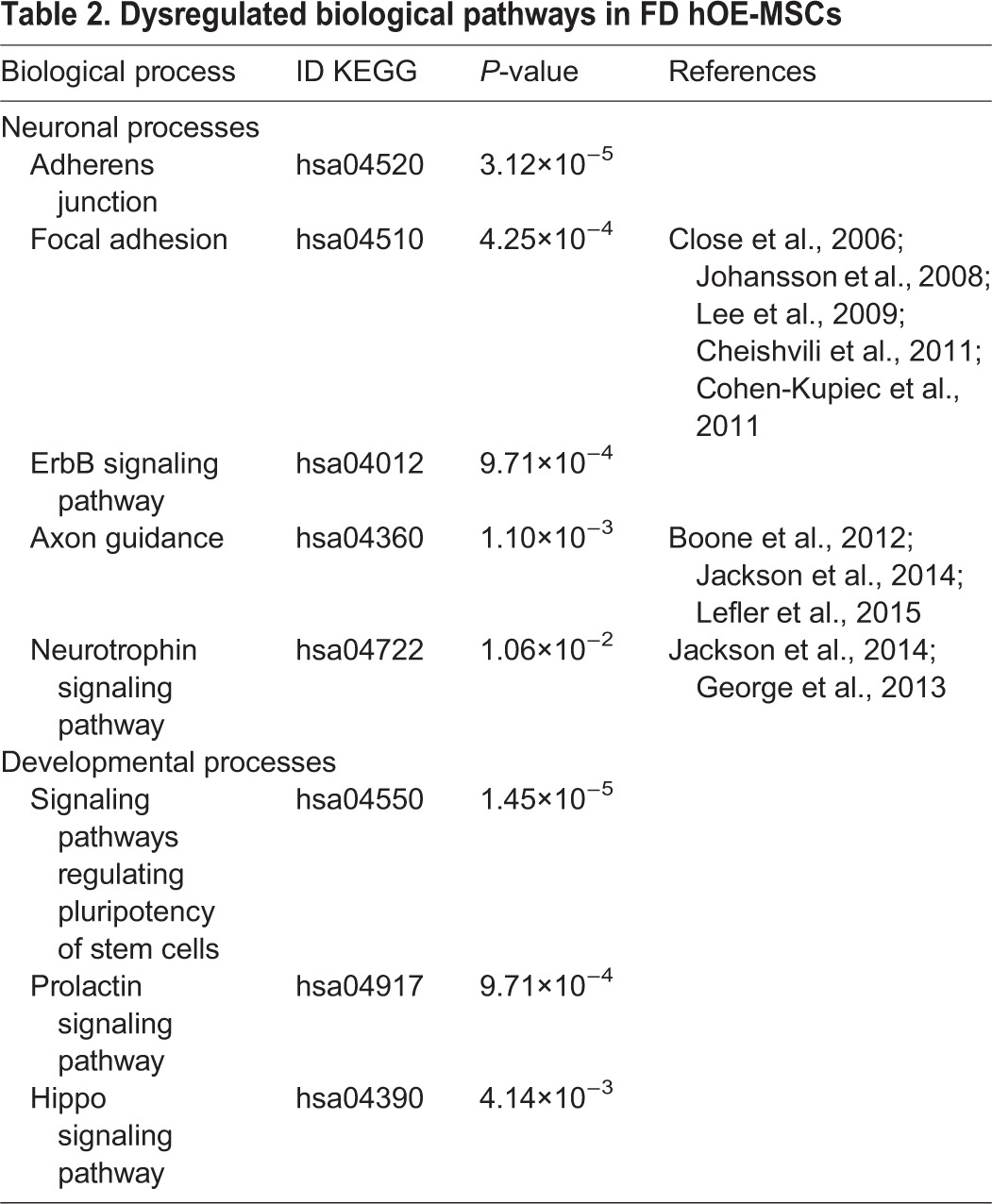
**Dysregulated biological pathways in FD hOE-MSCs**

### Establishment of dysregulated candidate miRNA:mRNA couples

To better understand the implications of dysregulated miRNAs within these different signaling pathways, we searched for their target mRNAs within the TarBase v7.0 database for validated mRNA–miRNA interactions and used the ComiR software for *in silico* predictions (Coronnello and Benos, 2013; Vlachos et al., 2015a). These tools allowed us to generate lists of target mRNAs determined through either experimental observations or computer algorithm predictions. We tested these ‘validated’ and ‘predicted’ mRNAs lists previously generated by genome-wide transcriptome data from several FD cell models (Boone et al., 2012; Cheishvili et al., 2007; Close et al., 2006; Cohen-Kupiec et al., 2011; Lee et al., 2009; Lefler et al., 2015) by selecting the target mRNAs that have antagonist expression variations with their corresponding miRNAs. Our analysis revealed nearly 235 mRNAs differentially expressed in FD cells and targeted by the dysregulated miRNAs in FD compared to control hOE-MSCs (Table S4). More than half of these mRNAs were related to nervous system pathways, thereby highlighting the potential involvement of these specific mRNAs in FD pathology (Table 3; mRNAs shaded in gray on Table S4).

**Table 3.**
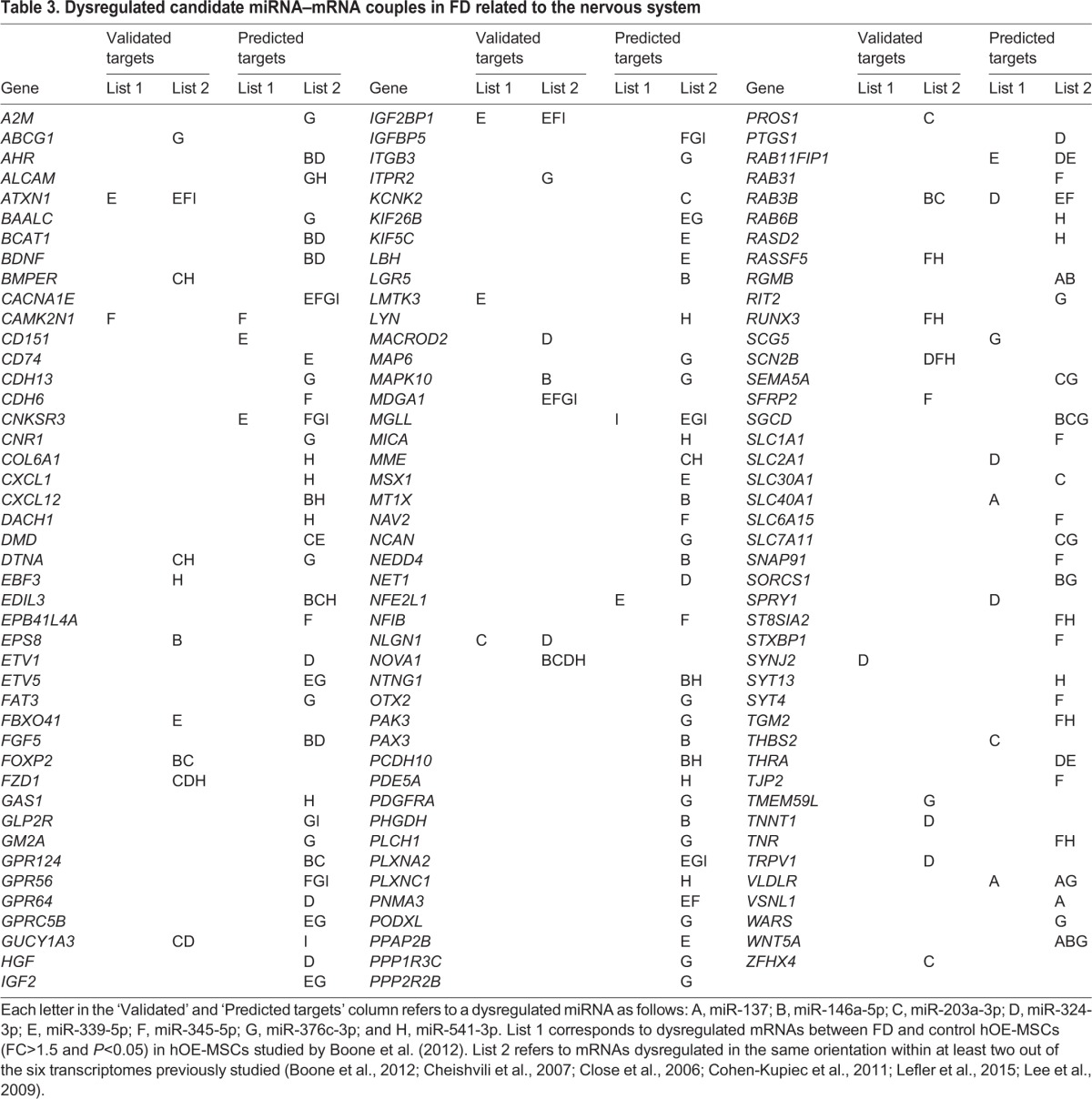
**Dysregulated candidate miRNA–mRNA couples in FD related to the nervous system**

Interestingly, we also found that four out of nine miRNAs differentially expressed in FD cells could potentially target the neuron-specific splicing factor *NOVA1* (Table S4), suggesting that NOVA1 could play a role as a regulatory factor in FD.

### Screening validation by individual RT-qPCR

**Fig. 2.**
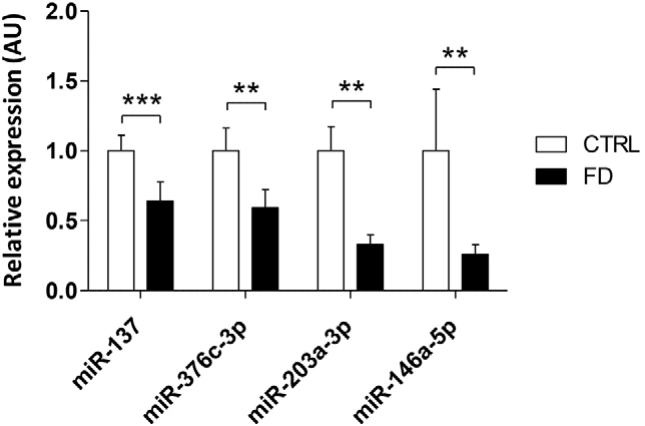
**Candidate miRNAs validation by RT-qPCR.** Histograms represent the mean±s.e.m. value of hsa-miR-137, hsa-miR-376c-3p, hsa-miR-203a-3p and hsa-miR-146a-5p relative expression level in two series of biological replicates from the first microfluidics arrays (five control and five FD hOE-MSCs) and the second arrays (four healthy control and four FD hOE-MSCs), respectively normalized by hsa-miR-320a (for both hsa-miR-137 and hsa-miR-376c-3p), hsa-miR-330-3p and hsa-miR-324-5p. The relative expression was calculated with reference to control samples (set at 1). **P<0.01; ***P<0.001 (linear mixed model test).

To validate our microarray findings, individual RT-qPCR was performed for the four dysregulated miRNAs targeting *NOVA1* using 18 samples (the ten used for the first microfluidics arrays and the eight for the second screen). We validated a significant underexpression in FD compared to control hOE-MSCs for all four miRNAs: miR-137, miR-376c-3p, miR-203a-3p and miR-146a-5p (Fig. 2). Consistent results found from miRNA screening and RT-qPCR reinforced the need for subsequent studies to explore the role of these dysregulated miRNAs in the nervous system development and function.

### Dysregulation of the neuron-specific splicing factor NOVA1

**Fig. 3.**
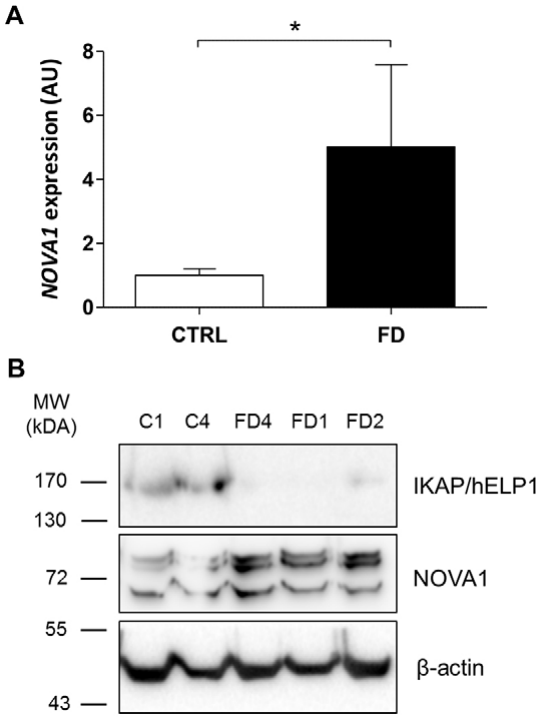
**Dysregulated NOVA1 expression in FD hOE-MSCs.** Histograms (A) represent the mean±s.e.m. level of expression of *NOVA1* mRNA in two series of biological replicates from the first (five control and five FD hOE-MSCs) and the second screening (four healthy control and four FD hOE-MSCs) determined by relative RT-qPCR. *WDR59* was used as the normalization gene. The relative expression in FD samples was calculated with reference to control samples (set at 1). *P<0.05 (linear mixed model test). Western blotting (B) shows the expression level of NOVA1 and IKAP proteins in two control and three FD samples. The β-actin expression level was used as a reference protein.

In order to study the role of the splicing factor NOVA1 in FD pathophysiology, we first decided to validate its differential expression and transcriptional and translational features in FD compared to control hOE-MSCs. We analyzed total RNA from the same previous 18 samples. As shown in Fig. 3A, *NOVA1* mRNA was significantly overexpressed by ∼5 times in FD compared to control cells. Accordingly, we demonstrated a similar increase of NOVA1 protein level in FD compared to control cells by western blotting (Fig. 3B, middle panel). We could also correlate low levels of WT *IKBKAP* mRNA (Fig. 1; Fig. S3) to a very weak IKAP protein signal in FD cells compared to control hOE-MSCs (Fig. 3B, upper panel). The observed dysregulation of NOVA1 in FD highlights the likely importance of this splicing factor in FD disease.

### A miRNA as a regulatory link between NOVA1 and IKAP

To assess the impact of NOVA1 in FD pathology, we attempted to restore correct expression level of this factor in FD cells. By modulating a dysregulated miRNA that was predicted to target *NOVA1*, we would be able to indirectly visualize an interaction between the modulated miRNA and *NOVA1*, as well as induce variations in the levels of *IKBKAP* and IKAP expression.

The NOVA1 factor was targeted by four dysregulated miRNAs in FD (Table S4). To choose the best miRNA for experimental expression variation, we selected one that exhibited the lowest level of expression in FD cells (Table 1; Fig. S2) and the highest rate and number of target mRNAs involved in nervous system function (genes shaded in gray on Table S4), and therefore further focused our study on the miR-203a-3p–*NOVA1* relationship.

**Fig. 4.**
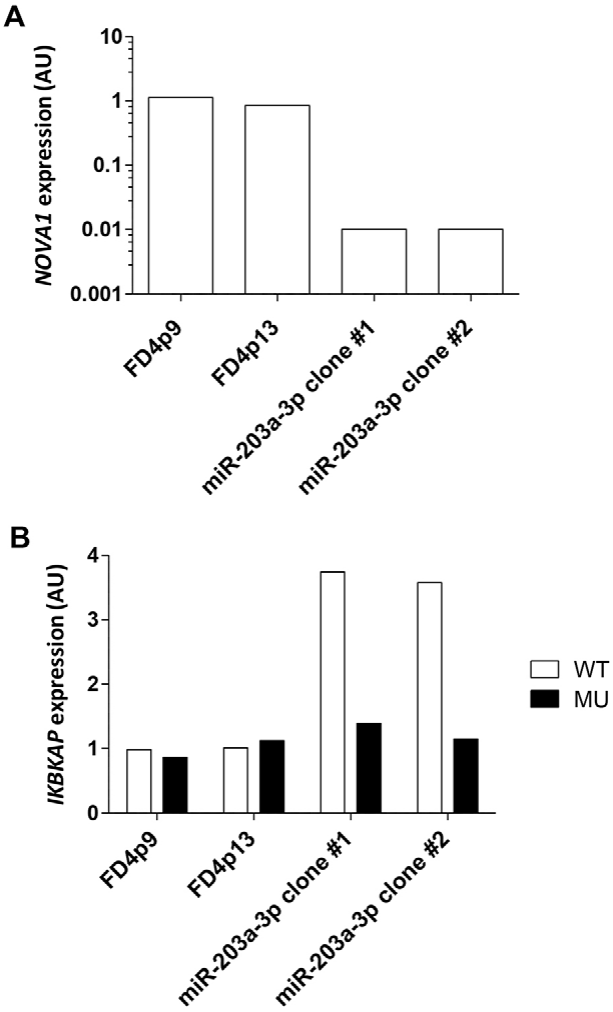
**miR-203a-3p overexpression induces antagonistic effects on *NOVA1* and *IKBKAP* WT transcripts levels.** Histograms represent the level of expression of *NOVA1* mRNA (A) and *IKBKAP* transcripts (B) in two primary cultures of FD hOE-MSCs at different cell passages (p9 and p13) and two FD clones (#1 and #2) obtained after transduction with miR-203a-3p lentiviral constructs, as determined by RT-qPCR. *WDR59* was used to normalize mRNA expression. The expression of each gene was determined relative to the median value of non-transduced FD hOE-MSCs (set at 1).

**Fig. 5.**
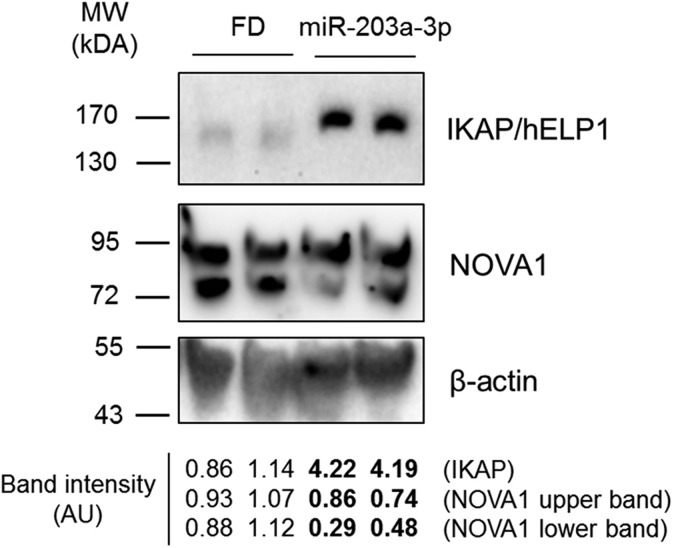
**miR-203a-3p overexpression downregulates NOVA1 and upregulates IKAP protein levels.** Western blotting shows expression level of IKAP and NOVA1 proteins in one culture of FD hOE-MSCs and one FD clone overexpressing miR-203a-3p. β-actin expression level was used as a housekeeping protein. A quantification of the band intensity is shown.

To modulate NOVA1, one culture of FD hOE-MSCs was transduced with the pLenti6/V5-DEST/miR-203a-3p construct to generate several clones. Transduction of lentivirus expressing miR-203a-3p in FD cells induced efficient overexpression (by ∼1000-fold) compared to non-transduced controls (Fig. S4). As expected, such an overexpression of miR-203a-3p induced a drastic decrease of the *NOVA1* mRNA expression level (Fig. 4A). Moreover, we observed a concomitant effect on *IKBKAP* mRNA aberrant alternative splicing, resulting in an increase by 3–4-fold of *IKBKAP* WT exclusively (Fig. 4B). Interestingly, we also found that miR-203a-3p overexpression induced a reduction of NOVA1 protein levels (Fig. 5, middle panel), which is also associated with an important increase of IKAP expression (Fig. 5, upper panel). These results underscore a link between miR-203a-3p, NOVA1 and IKAP.

## DISCUSSION

Genome-wide investigations of transcriptional variations are commonly used to identify signatures of neurodevelopmental and neurodegenerative disorders (Cooper-Knock et al., 2012; Mufson et al., 2006; Parikshak et al., 2015). In the last decade, within the RNA world, miRNAs appeared to be essential regulators of most biological processes, through interaction with various mRNAs.

In this study, we assumed that *IKBKAP* alternative splicing is essential to trigger the cascade of molecular events leading to FD pathophysiology but at a higher level, some distortion of the physiological miRNAs network could also play a role. Indeed, we learned from previous attempts to model FD in animals that a very narrow window of *IKBKAP* expression at specific locations during development separates a viable model with symptoms close to FD patients from embryonic lethality (Abashidze et al., 2014; Chen et al., 2009; Creppe et al., 2009; Dietrich et al., 2012, 2011; George et al., 2013; Hunnicutt et al., 2012; Jackson et al., 2014; Morini et al., 2016). Therefore, for many mutations affecting important genes or biological pathways, we surmised that adaptive mechanisms are engaged during embryonic and fetal development to compensate for the loss of function resulting from a decrease in IKAP, as well as the catalytically active Elongator complex. Among the compensatory mechanisms, the modulation of miRNA activities represent a powerful way to modify the level of expression of tens to hundreds of proteins so that FD cells could survive to the burden of a severe mutation (Ebert and Sharp, 2012; Hornstein and Shomron, 2006; Posadas and Carthew, 2014). For this reason, we profiled miRNA expression using control and FD hOE-MSCs to reveal specific miRNA alterations in FD. To our knowledge, this is the first study to analyze human miRNAs signature in the FD context, and to suggest a feedback mechanism by which compensatory mechanisms would result in modulating the activity of a factor in front line to direct tissue-specific alternative mRNA splicing in the nervous system.

Several studies have demonstrated the viability of using hOE-MSCs for the study of neurological disorders (Feron et al., 2015; Mackay-Sim, 2012; Matigian et al., 2010). FD-patient-derived cellular tissue obtained by a non-invasive method is an informative model, recapitulating different aspects of *IKBKAP* gene expression specific to the FD pathology (Boone et al., 2012, 2010). Moreover, two studies published in the last few years have shown that hOE-MSC samples might reflect the brain miRNA signature (Mor et al., 2013; Nguyen et al., 2016). To validate the miRNA expression profiles obtained in control hOE-MSCs, we compared them to the ones from control olfactory stem cells of Mor and colleague's study (Fig. S5) (Mor et al., 2013). Although experiments were conducted with different biopsies and in distinct laboratories, miRNA expression patterns were quite similar, indicating the reliability of our study and the pertinent choice of hOE-MSCs as a cellular model to characterize miRNA expression patterns.

Prior to performing our microfluidics arrays experiments, we hypothesized that there might be a unique miRNA signature in FD pathology. To identify the specific FD signature, we calculated the relative expression of each miRNA between FD and control samples by normalizing data to reference miRNAs. In fact, we had previously observed, using peripheral blood samples from patients with major depression, that small RNAs commonly used for target miRNA normalization, such as RNU44, RNU48 and U6 snRNA, were expressed at higher orders of magnitude compared to the majority of miRNAs (Belzeaux et al., 2012). In hOE-MSCs, RNU44, RNU48 and U6 snRNA were among the most highly expressed RNAs (Table S2), whereas 60% of our analyzable miRNAs were much less expressed and could be detected only after an additional 5 to 12 Ct by RT-qPCR. As additional support for why we chose to select specific miRNAs for normalization of our experimental cell type, another study had previously demonstrated that reference miRNAs specific to each cell type have increased stability compared to the small RNAs commonly used for normalization (Peltier and Latham, 2008).

In accordance with our initial assumption that there is a specific miRNA expression profile in FD, we found several dysregulated miRNAs in FD compared to control hOE-MSCs. Interestingly, the dysregulated miRNA expression pattern appeared to be similar to the mRNA expression pattern we had previously described (Boone et al., 2012), with a predicted association between discriminant miRNAs and mRNAs. Concordance between these two works suggests a potential role of miRNAs as therapeutic targets to regulate dysregulated mRNAs expression. Indeed, some studies had previously examined the involvement of a miRNA network in neurodegenerative diseases (Hebert and De Strooper, 2009; Lau et al., 2013; Ubhi et al., 2014) and demonstrated the possibility to target miRNAs to treat neurodegenerative disorders (Gupta et al., 2015; Weinberg and Wood, 2009).

The detailed analysis of discriminating miRNAs revealed that none of our dysregulated miRNAs directly target the *IKBKAP* gene, suggesting existence of an indirect regulation pathway in FD. Nevertheless, two miRNAs were similarly dysregulated in our two microfluidics arrays experiments, hsa-miR-345-5p and hsa-miR-137, emphasizing a concordance of our two studies. Moreover, we found that one of our discriminant miRNAs, hsa-miR-146a, was similarly dysregulated in another study conducted on olfactory stem cells about autism spectrum disorders (Nguyen et al., 2016), and might be linked with diabetic neuropathy, suggesting a possible common regulation mechanism in various neurodegenerative pathologies. To go further in the validation of miRNAs signature in FD, we searched to establish a relationship between other dysregulated miRNAs and neurodegenerative pathologies. In the literature, some miRNAs dysregulated in FD are well characterized and are known to be associated with the nervous system function. For example, miR-17-5p seems to regulate amyloid precursor protein expression and therefore to be related to Alzheimer's pathology (Hebert et al., 2009). Regarding the miRNA we chose to focus on, miR-203a-3p, it is mainly known to play a role in epithelial differentiation and tumorigenesis, but recent work has established that this miRNA can modulate the expression level of *NEGR1*, which encodes a protein implicated in neurite outgrowth during neuronal development (Benaich et al., 2014; Kaur et al., 2016; Taube et al., 2013; Yi et al., 2008). Therefore, our data linking miR-203a-3p with NOVA1 would corroborate miR-203a-3p contribution in the nervous processes.

Furthermore, we used an *in silico* approach to link miRNAs to neurological processes, such as neurotrophins signaling pathway or axon guidance (Table 2). Alterations of neuronal pathways were similarly observed in hESC-derived PNS neurons from control and FD patients, and can be related to the incomplete development of the nervous system observed in FD patients (Abashidze et al., 2014; Axelrod, 2004; Gardiner et al., 2008; Lefler et al., 2015; Nosrat, 1998). Interestingly, among the intracellular processes (Table S3) that were dysregulated in FD cells, the FoxO signaling pathway could be related to vascular network abnormalities previously observed in *IKBKAP* knockout mice because the transcription factor FoxO3 has been implicated in angiogenesis (Chen et al., 2009; Koenig et al., 2014). In addition, a recent paper has established a relationship between a deficit in the regulation of actin cytoskeleton and aberrant axon trafficking observed in peripheral neuropathies (Tourtellotte, 2016). In parallel, we predicted *in silico* sets of mRNAs targeted by dysregulated miRNAs. Importantly, we were able to identify some of the same targets as in our previously published genome-wide expression study (Boone et al., 2012). Many of these candidate genes are associated with neural development and neurological disease. *BDNF* is involved in neuronal growth and differentiation that is crucial for the pattern of the developing peripheral nervous system (Kasemeier-Kulesa et al., 2015), and is dysregulated in Alzheimer's disease, Parkinson's diseases, schizophrenia and depression (Angelucci et al., 2005; Murer et al., 2001). *FOXP2* plays a role in neurogenesis and is involved in language and speech disorders (Chiu et al., 2014; Roll et al., 2010; Tsui et al., 2013) *SEMA5A*, which encodes members of the semaphorin family, has been implicated in axonal guidance and is a candidate gene in autism spectrum disorder (Cheng et al., 2013; Melin et al., 2006). *RUNX3* is essential to peripheral neuron development and has been previously related to insufficient production of nociceptors and thermoreceptors in FD (George et al., 2013; Nakamura et al., 2008). *TRPV1* encodes for a key cation channel in nociception and is implicated in some neurological disorders (Jendryke et al., 2016; Madasu et al., 2015). Moreover, we have previously validated that the gelsolin gene, *GSN*, which has previously been shown to be downregulated in several animal models linked to FD (Chen et al., 2009; Close et al., 2006) is a target of hsa-miR-339-5p. Interestingly, amongst the target genes, *NOVA1* was the only neuron-specific splicing factor previously shown to be dysregulated in two others studies (Boone et al., 2012; Lee et al., 2009). This gene is a tissue-specific factor contributing to alternative splicing of many genes in the brain (Jelen et al., 2007; Jensen et al., 2000; Racca et al., 2010). It has been suggested to play a role in proper synaptic development and function (Ruggiu et al., 2009). Moreover, this splicing factor is known to regulate neuronal miRNA function by interacting with Ago proteins, suggesting strong interconnectivity between the NOVA1 and miRNA network (Storchel et al., 2015). In this paper, we predicted that *NOVA1* could be targeted by four dysregulated miRNAs in FD versus control hOE-MSCs, suggesting that regulation of *NOVA1* by miRNAs could modulate *IKBKAP* alternative splicing. Indeed, underexpression of NOVA1, achieved by modulating expression of a miRNA targeting this gene, resulted in correction of *IKBKAP* aberrant alternative splicing, as well as improved IKAP expression level. This supports the existence of a direct or indirect interaction, between NOVA1 and IKAP. This potential link is reinforced by the presence of several NOVA1 target sequences (pyrimidine-CA- pyrimidine clusters) in *IKBKAP* RNA (Ule et al., 2006). Overall, our results suggest that the splicing factor NOVA1 could be an important regulatory component contributing to FD etiology. This possibility is reinforced by the fact that neurodegenerative diseases could be essentially induced by alterations of the splicing process (Ling et al., 2015; Smith et al., 2011).

To strengthen our work, it will be interesting to explore miRNA alterations in the sensory neurons that die from FD pathogenesis. Some studies have been previously conducted on IKAP-deficient neurons and revealed newly affected processes in FD such as defects in post-migratory sensory and sympathetic neuron survival, target tissue innervation and TrkA neuron development (George et al., 2013; Jackson et al., 2014). Therefore, these neuronal models could lead to new understandings about miRNA network in FD. In addition, our study could be complemented by the examination of combined miRNA and mRNA patterns in integrated models, such as animal models. Indeed, isolated *in vitro* cultured cells cannot recapitulate all existing interactions occurring within the *in vivo,* complex, three-dimensional environment of a living human. Three animal models of FD have been recently established that recapitulate most of the genetic and phenotypic FD defects (Dietrich et al., 2012, 2011; Morini et al., 2016). Moreover, the effects of some molecules able to modulate *IKBKAP* aberrant splicing, such as kinetin and its derived compounds, on miRNA and mRNA profiles could be studied in animal models to determine genuine effects of these corrective drugs in the context of a complex organism (Shetty et al., 2011). Although there are limitations concerning the use of cellular models to question the direct application of the newly identified candidate miRNAs as therapeutic targets (Rukov and Shomron, 2011), previous studies on neurodegenerative disorders have demonstrated miRNA dysregulations identified using cellular models could lead to isolate new therapeutic targets able to modify phenotypic features in animals (Weinberg and Wood, 2009).

## MATERIALS AND METHODS

### Cell culture of hOE-MSCs

The cultures of hOE-MSCs derived from five FD patients and five healthy controls have been previously described (Boone et al., 2012, 2010), and were maintained in DMEM/Ham's F12 medium (Life Technologies, Carlsbad, CA) containing 10% fetal bovine serum (FBS) (PAA, Pasching, Austria) and 0.1% gentamicin (Life Technologies, Gibco, Grand Island, NY) at 37°C in the presence of 5% CO_2_. Adherent cells were detached after washes with phosphate-buffered saline (PBS) (Life Technologies, Gibco, Grand Island, NY) by addition of Trypsin-EDTA (0.05% Trypsin, 0.5 mM EDTA, Life Technologies, Gibco, Grand Island, NY) during 5 min at 37°C. Cells pellet was recovered after a centrifugation of 5 min at 300 ***g***.

### RNA extraction

Total RNA was purified with the mirVana miRNA isolation kit (Life Technologies, Ambion, Austin, TX) according to the manufacturer's protocol, without any enrichment in miRNA, and submitted to DNase treatment (DNA-free™ kit, Life Technologies, Ambion, Austin, TX). RNA concentration was determined using a nanodrop ND-1000 spectrophotometer (Thermo Scientific, Waltham, MA). RNA integrity was assessed by use of the RNA integrity number (RIN) score determined on an Agilent 2100 Bioanalyzer (Agilent Technologies, Santa Clara, CA) with the RNA 6000 Nano kit (Agilent Technologies, Santa Clara, CA).

### miRNA quantification by microfluidics arrays

120 ng of each total RNA sample was reverse transcribed into cDNA using the TaqMan MicroRNA Reverse Transcription kit (Life Technologies, Applied Biosystems, Foster City, CA) in combination with the stem-loop Megaplex™ RT primers, Human Pool A v2.1 (Life Technologies, Applied Biosystems, Foster City, CA), with preamplification and according to manufacturer's recommendation. 16 ng of the resulting cDNA was combined with TaqMan Universal PCR Master Mix II with no uracil N-glycosylase (Life Technologies, Applied Biosystems, Foster City, CA) and 100 µl of mix was loaded into each port of the 384 wells TaqMan Human MicroRNA Array A v2.0 (Life Technologies, Applied Biosystems, Foster City, CA). PCRs were run on an ABI PRISM 7900HT thermocycler under the following conditions: 2 min at 50°C, 10 min at 95°C, 40 cycles of 15 s at 95°C and 1 min at 60°C. Raw Ct values were calculated using the RQ Manager software with manual baseline settings for each miRNA. Only those miRNAs that were detectable (Ct<32 in >20% of the hOE-MSCs samples) were subsequently analyzed. The relative expression level of each miRNA was quantified as 2^−ΔΔCt^ (Livak and Schmittgen, 2001), and therefore normalized based on the expression level of a reference miRNA, exhibiting a stable level of expression among all samples and selected with DataAssist and NormFinder softwares, and a calibrator sample (defined by the mean Ct of the control samples). In order to normalize the miRNAs with reference miRNA exhibiting a proximal expression level, five windows of expression levels (Ct<21; 21<Ct<24; 24<Ct<27; 27<Ct<30; Ct>30) were defined in which a specific reference miRNA was selected.

The same protocol was applied a second time on biological replicate total RNA from four healthy control and four FD hOE-MSCs. For this additional round of microfluidics arrays, the eight RNA samples was reverse transcribed in cDNA then directly amplified by qPCR, without a preamplification step. The threshold to select analyzable miRNAs was fixed at Ct<35 in >25% of samples, and miRNAs were classified into five categories of expression levels (Ct<24; 24<Ct<27; 27<Ct<30; 30<Ct<33; Ct>33) to choose adequate reference miRNAs.

### Individual assays for miRNA expression quantification

10 ng of total RNA sample was reverse transcribed with the TaqMan MicroRNA Reverse Transcription (Life Technologies, Applied Biosystems, Foster City, CA). 1 ng of the resulting cDNA was combined with a TaqMan universal PCR Master Mix II with no uracil N-glycosylase (Life Technologies, Applied Biosystems, Foster City, CA) and PCR was performed in triplicate on an ABI PRISM 7900HT thermocycler according to the manufacturer's recommendation. For each tested miRNA, specific primer couples and probes were selected using the web portal of the manufacturer (Life Technologies, Carlsbad, CA): hsa-miR-137 (ID 000593), hsa-miR-146a-5p (ID 000468), hsa-miR-203a-3p (ID 000507), and hsa-miR-376c-3p (ID 002122). The relative expression level of each miRNA was calculated using the 2^−ΔΔCt^ method, in which each miRNA is quantified relative to the expression of one reference miRNA, chosen among hsa-miR-324-5p (ID 000539), hsa-miR-320a (ID 002277) and hsa-miR-330-3p (ID 000544) according to the target miRNA level of expression, and a calibrator sample.

### Quantification of mRNA expression

1 µg of total RNA was subjected to reverse transcription using the High-Capacity cDNA Reverse Transcription kit (Life Technologies, Applied Biosystems, Foster City, CA). PCR was performed in duplicate with the TaqMan Fast Advanced mix (Life Technologies, Applied Biosystems, Foster City, CA) on 50 ng of the resulting cDNA, using an ABI PRISM 7900HT thermocycler under the following conditions: 2 min at 50°C, 20 s at 95°C, 40 cycles of 1 s at 95°C and 20 s at 60°C. Primers hELP1 ex19F, hELP1 ex21R, and probe P-WTELP1 ex20R were used for detection of *IKBKAP* transcripts containing exon 20, and primers hELP1 ex19-21F, hELP1 ex21-22R, and probe P-MUELP1 ex21F were used for detection of *IKBKAP* transcripts skipping exon 20 (Boone et al., 2010). Primers and TaqMan probes assays from Applied Biosystems were used to determine the level of expression of *NOVA1* transcripts (Hs00359592_m1) and the reference gene to normalize the data, *WDR59* (Hs00226608_m1). Results were calculated using the 2^−ΔΔCt^ method.

### Statistical analysis

After each miRNA screening, to select differentially expressed miRNAs in each group comparison, we determined miRNAs presenting a FC superior to 1.3, given that there is no scientific consensus for a FC threshold, then coupled this method with statistical analysis using parametric Student's *t*-test to compare the FC between FD patients and healthy controls using IBM SPSS Statistics v20 software. For individual validation of miRNA dysregulation, linear mixed models were used to study the FC significance between FD patients and controls. The linear mixed model was also used to analyze *NOVA1* RT-qPCR data, using R software. All statistical tests were made with a threshold *P*-value of 0.05.

### *In silico* biological pathways and target prediction

Signaling pathways linked to dysregulated miRNAs were determined using the DIANA-mirPath v3.0 web server that utilizes the DIANA-TarBase v7.0 validated database and predicted miRNA targets (in CDS or 3′-UTR regions) provided by the microT-CDS v5.0 and TargetScan algorithm. The software was used in the union genes mode, the false discovery rate (FDR) was enabled and various thresholds were set at 0.05 for the *P*-value, 0.8 for the microT and 0.1 for the TargetScan Conservation Score. For each dysregulated miRNA, validated mRNA targets were provided by the DIANA-TarBase v7.0 database (Vlachos et al., 2015a), and predicted mRNA targets were identified by using the combinatorial miRNA target (ComiR) prediction tool with the significance score set above 0.85 (Coronnello and Benos, 2013; Coronnello et al., 2012). Then, ‘validated’ and ‘predicted’ mRNAs targets were compared to lists of discriminated mRNAs from a previous transcriptome analyses (Boone et al., 2012), with list #1 representing dysregulated mRNAs in FD versus CTRL hOE-MSCs [FC>1.5 and *P*<0.05, (Boone et al., 2012)] and list #2 corresponding to dysregulated mRNAs found in two transcriptome analyses out of six of other cell types (Boone et al., 2012; Cheishvili et al., 2007; Close et al., 2006; Cohen-Kupiec et al., 2011; Lefler et al., 2015; Lee et al., 2009). Only the miRNA–mRNA couples with antagonist expression variation were retained.

### Lentiviral miRNA overexpression

MiR-203a-3p oligonucleotides (FW 5′-TGCTGCTAGTGGTCCTAAACATTTCACGTTTT GGCCACTGACTGACGTGAAATGTAGGACCACTAG-3′; REV 5′-CCTGCTAGTGGTC CTACATTTCACGTCAGTCAGTGGCCAAAACGTGAAATGTTTAGGACCACTAGC-3′) were purchased from Eurofins MWG Operon (Ebersberg, Germany). Lentiviral particles were obtained using the BLOCK-iT™ PolII miR RNAi Expression Vector kit with EmGFP (ThermoFisher Scientific, Invitrogen, Carlsbad, CA), according to the manufacturer's recommendation. In brief, small hairpin sequence corresponding to miR-203a-3p was cloned into the pLenti6/V5-DEST vector, which was then packaged into replication-incompetent lentiviral particles in HEK293FT cells by co-transfecting pLenti6/V5 plasmid with the ViraPower Packaging Mix. Viral particles were collected 72 h post transfection in the supernatant after a centrifugation of 5 min at 750 ***g*** at 4°C. One culture of FD hOE-MSCs was transduced with the pLenti6/V5-DEST/miR-203a-3p lentivirus for 24 h with Polybrene at 6 µg/ml (ThermoFisher Scientific, Invitrogen, Carlsbad, CA). Several clones were generated by limiting dilutions under blasticidin selection at 10 µg/ml (ThermoFisher Scientific, Invitrogen, Carlsbad, CA).

### Western blotting

For analyses by western blotting, hOE-MSCs were collected by trypsination, counted and then centrifuged for 5 min at 300 ***g***. Cell pellets were resuspended in 1× SDS loading buffer, heated at 95°C during 5 min, then each cell lysate was resolved by 8% SDS-PAGE and transferred onto a PVDF membrane (Thermo Scientific, Rockford, IL). After blocking membrane with 5% milk in PBS (Life Technologies, Gibco, Grand Island, NY) supplemented with 0.1% Tween-20 (Sigma-Aldrich, St. Louis, MO) (PBS-T), membrane was incubated at 4°C overnight with mouse monoclonal primary antibodies against IKAP diluted at 1:2000 (clone no 33, BD Biosciences, Franklin Lakes, NJ), anti-NOVA1 at 1:300 (cat. no WH0004857M7, Sigma-Aldrich, St. Louis, MO) or anti-β-actin at 1:10,000 (cat. no A2228, Sigma-Aldrich, St. Louis, MO) in 2.5% milk in PBS-T. Thereafter, membrane was then probed with mouse secondary antibodies diluted at 1:3500 (Sigma-Aldrich, St. Louis, MO) in 2.5% milk in PBS-T at room temperature for 45 min, and revealed by exposing pre-incubated membrane with ECL (ThermoScientific, Rockford, IL) using a G:BOX Chemi XT4 device (Syngene, Cambridge, UK). β-actin was used for normalization as the housekeeping protein.
